# UV radiation enhanced oxygen vacancy formation caused by the PLD plasma plume

**DOI:** 10.1038/s41598-018-27207-5

**Published:** 2018-06-11

**Authors:** F. V. E. Hensling, D. J. Keeble, J. Zhu, S. Brose, C. Xu, F. Gunkel, S. Danylyuk, S. S. Nonnenmann, W. Egger, R. Dittmann

**Affiliations:** 1 Peter Grüneberg Institut 7, Forschungszentrum Jülich, 52425 Jülich, Germany; 2 Carnegie Laboratory of Physics, SUPA, School of Science and Engineering, University of Dundee, Dundee, DD1 4HN United Kingdom; 3 Mechanical and Industrial Enginnering, University of Massachusetts, Amherst, MA 01003-2210 USA; 4 Chair for Technology of Optical Systems, RWTH Aachen University, Steinbachstr. 15, 52074 Aachen, Germany; 5 Institute of Electronic Materials (IWE2), RWTH Aachen University, 52074 Aachen, Germany; 6 Universität Bundeswehr München, 85577 Neubiberg, Germany

## Abstract

Pulsed Laser Deposition is a commonly used non-equilibrium physical deposition technique for the growth of complex oxide thin films. A wide range of parameters is known to influence the properties of the used samples and thin films, especially the oxygen-vacancy concentration. One parameter has up to this point been neglected due to the challenges of separating its influence from the influence of the impinging species during growth: the UV-radiation of the plasma plume. We here present experiments enabled by a specially designed holder to allow a separation of these two influences. The influence of the UV-irradiation during pulsed laser deposition on the formation of oxygen-vacancies is investigated for the perovskite model material SrTiO_3_. The carrier concentration of UV-irradiated samples is nearly constant with depth and time. By contrast samples not exposed to the radiation of the plume show a depth dependence and a decrease in concentration over time. We reveal an increase in Ti-vacancy–oxygen-vacancy-complexes for UV irradiated samples, consistent with the different carrier concentrations. We find a UV enhanced oxygen-vacancy incorporation rate as responsible mechanism. We provide a complete picture of another influence parameter to be considered during pulsed laser depositions and unravel the mechanism behind persistent-photo-conductivity in SrTiO_3_.

## Introduction

The field of transition metal oxides has opened various research opportunities over the last decade due to their manifold interesting properties (e.g. electronic and magnetic)^1,2^. As the importance of complex metal oxide research has grown, there has been a commensurate increase in importance of pulsed laser deposition (PLD). PLD has proved a versatile and powerful tool for the deposition and epitaxial growth of this class of multicomponent materials^2–4^.

The properties of these transition metal oxides are highly depended on their stoichiometry and the presence of point defects. Prominent examples are oxygen vacancy induced conduction^5–8^, and the suppression of donor doping by cation vacancies^9–16^. Deposition of high quality epitaxial oxide films is normally enabled by using oxide single crystal substrates. It has been shown that the point defect density of both, the thin film and the substrate, play a crucial role. The substrate can, for example, strongly contribute to the conductivity if a significant amount of oxygen vacancies is generated during growth^17–20^.

The point defect densities within thin film and substrate induced during PLD growth generally differ strongly from the expected equilibrium values due to the involved complex non-equilibrium processes. For SrTiO_3_ (STO), one of the most commonly used transition metal oxide substrates, the influence of various process parameters on the formation of oxygen vacancies has been investigated in great detail^20–26^. The formation of oxygen vacancies is especially relevant when working at low deposition pressures. Up to now, several reasons were found for the equilibrium exceeding formation of oxygen vacancies: the oxidization of the oxygen deficient thin film *via* the substrate resulting in the formation of oxygen vacancies in the substrate^21–23^, impinging species of the plasma plume resulting in an oxygen removal^24–26^, and even an influence of the applied measurement devices^20^.

While the effect of impinging species from the PLD plasma plume has been recognized and studied^24–26^, the possible effect of UV-radiation emitted by the plume has not been considered, although the plasma plume is known to emit UV-radiation^4^. Further UV radiation is known to induce persistent photo conductivity^27,28^, as well as to enhance the oxygen incorporation rate^29–33^, in STO.

One obstacle with respect to evaluate the influence of UV-radiation during PLD growth is the problem of separating its influence from that of the impinging species within the plume. In order to allow such a separation, we designed a new sample holder, allowing UV-radiation to reach the sample, but not the impinging species. STO samples are processed in this new holder at typical low pressure PLD conditions, one sample with an ignited plume and, as a reference, one without. Hall measurements show an increase in charge carrier concentration in STO samples exposed to the plume UV-radiation, compared to the reference sample. Cross sectional scanning Kelvin probe microscopy (SKPM) measurements revealed a difference in the carrier concentration profile between the two samples. Variable energy positron annihilation lifetime spectroscopy (VEPALS) measurements detect Ti vacancy – oxygen vacancy complexes (*V*_*Ti*_*V*_*O*_) in the near-surface region of both types treated STO samples.

## Results

**Figure 1:**
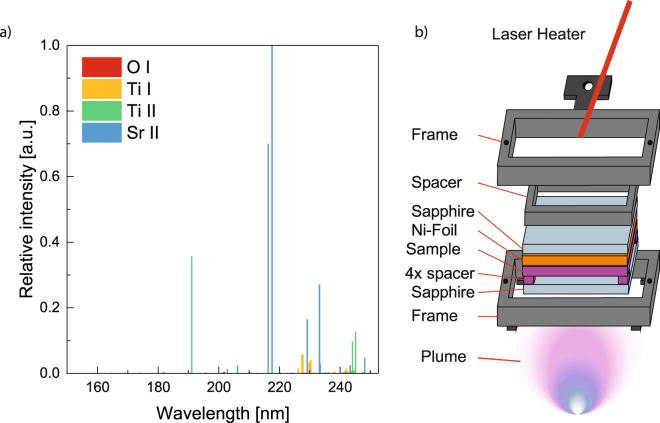
(**a**) Simulated spectra in the UV regime for an STO plasma plume. (**b**) Sample holder designed to separate the influence of the UV-radiation and the impinging species from the plasma plume during PLD. The front sapphire window is transparent to UV-radiation, but prevents the ablated atomic and molecular species of the plume from reaching the STO sample.

The first step towards an understanding of the role of UV radiation during PLD processes is to characterize the spectrum. The UV-spectrum of the plasma plume resulting from the PLD ablation of an STO target is simulated using *Saha*-*LTE*. To obtain the ionization levels from the *NIST* atomic spectra database needed for this simulation the electron temperature and density in the plasma plume are required^34^. The electron temperature (*kT*) in the plasma plume can be derived from the power density of the used ablation laser, $(1\pm 0.5)\times {10}^{8}\tfrac{{\rm{W}}}{{{\rm{cm}}}^{2}}$, using the corresponding equation by Schriever *et al*.^35^, to be 0.885 ± 0.135 eV. The electron density in the plasma plume is estimated to be ≈1 × 10^18^ cm^−3^ as given by Gilgenbach *et al*.^36^. The simulated UV-radiation spectrum of the plasma plume resulting from ablation of an STO target is shown in Fig. 1(a). Strong emission lines at ≈215 nm due to strontium are observed, and will contribute considerable intensity to the emitted UV radiation.

The sample holder designed for this study is shown in Fig. 1(b), the laser plume is blocked by a sapphire window which is transparent to UV-radiation ≥150 nm^37^. The STO sample is separated from the window by four 1 × 1 × 0.5 mm^3^ STO spacers, this exposes the sample surface to a gas exchange volume. The STO sample is mounted into the holder with a Ni-foil placed between the rear of the sample and a second sapphire window. The foil acts as an absorber for the infrared diode laser heater which provides precise control of the sample temperature. The back sapphire window enables transmission of the IR-radiation and prevents the ablation of the Ni-foil. Finally a spacer is placed between the second window and the holder frame to tighten the whole stack and improve heat transfer.

Experiments were performed on TiO_2_-terminated STO samples^38^ heated to 800 °C and in chamber pressure of 10^−5^ mbar oxygen for one hour. The sample temperature is rapidly quenched to room temperature by switching off the laser heater (cool down time ≈65 s). These are typical conditions for low pressure growth of oxide thin films, for which we showed in our earlier studies no reoxidization appears even after one hour^20^. At the same time, quenching restores as best as possible the defect state obtained immediately after the growth, and, hence allows to decouple growth phenomena from thermodynamic phenomena, such as reoxidation kinetics addressed in our previous work^19^. The effect of the UV-radiation from the plasma plume was then investigated by ablating an STO single crystal target using a excimer laser with a wavelength of 248 nm and a 5 Hz pulse repetition rate. Laser ablation was maintained for the 1 h period and the STO target was rotated at 5 rpm. Samples were prepared with and without the presence of the laser plume.

The sheet carrier concentration, *n*_*S*_, of the processed STO samples, with and without exposure to UV-irradiation, were measured using a Hall measurement system. Measurements were performed immediately following processing and after 50 days of storage in air for at least four equally treated samples. The averaged results including the standard deviation are shown in Table 1. Both the UV-irradiated and the non-irradiated samples exhibit a high sheet carrier concentration immediately after preparation. It is well known that annealing of STO at 10^−5^ mbar can result in the reduction of the samples^20,39–41^. The sheet carrier concentration of the UV-irradiated samples, however, is approximately twice that of the non irradiated samples immediately after processing. More strikingly, after 50 days the difference between the two types of samples becomes marked. The sheet carrier concentration of the UV-irradiated samples is unchanged, while the concentration in the non-irradiated samples has fallen below the measurement limit (<10^10^ cm^−2^).

**Table 1:** Sheet carrier concentration calculated from Hall effect measurements for low pressure annealed UV-irradiated and non-UV-irradiated STO single crystal samples. The samples were measured immediately after processing and after a further 50 day storage in air. The error indicates the standard deviation obtained from measuring at least four equally treated samples.

Sample	*n*_*S*_ (immediate)	*n*_*S*_ (50 d)
UV irradiated	(5 ± 1) × 10^17^ cm^−2^	(5 ± 1) × 10^17^ cm^−2^
Non UV irradiated	(2 ± 0) × 10^17^ cm^−2^	<10^10^ cm^−2^

A similar behavior was observed for samples treated at different oxygen pressure. Samples processed at 10^−4^ mbar oxygen showed the same behavior as samples processed at 10^−5^ mbar with a 50% lower sheet carrier concentration. Samples processed at 10^−6^ mbar showed an increased carrier concentration and no time dependence during storage in air. It can thus be assumed that their whole bulk was reduced. Samples processed at 10^−3^ mbar, however, showed no signs of reduction, which is in accordance with our previous results^19^. In the following we will exemplary discuss the samples processed at 10^−5^ mbar as they show the most pronounced differences between irradiated and non-irradiated samples, while having a high initial sheet carrier concentration.

In order to deliminate our results from conductivity contributions generated by photo-induced carriers only, we have carried out reference experiments at room temperature and 10^−5^ mbar, where photo-induced carriers may be generated by UV irradiation, while the kinetics of ionic defect formation limits the photo-induced formation of oxygen vacancies to the very-surface region. This is of high interest, as a reduction of STO is commonly also observed for room temperature depositions^25,42^. However no reduction of the substrates was observed without the presence of impinging species.

The higher sheet carrier concentration and its persistence after 50 days storage provide evidence that the STO sample defect structure, in particular the oxygen vacancy concentration, is altered significantly by the UV-irradiation from the plasma plume during low pressure processing.

**Figure 2:**
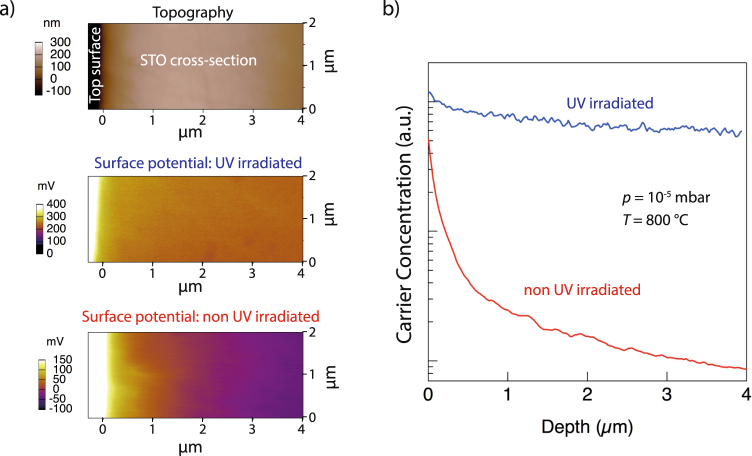
(**a**) The surface potentials below the STO surface (0 *μ*m) measured by cross sectional SKPM including a topography image for orientation. (**b**) The local carrier concentration with depth below the STO surface determined *via* the surface potential. Samples exposed to UV-irradiation during low pressure anneal (blue), and samples low pressure annealed but without UV exposure (red).

The question arises, whether the carrier concentration is homogeneously distributed through the volume of the sample, or if a concentration profile results. To investigate the depth dependence of the carrier concentration cross-sectional SKPM measurements were performed 10 days after processing. The results are shown in Fig. 2. Figure 2(a) shows the topography image for orientation (top) and the surface potentials for the UV irradiated sample (center) and the non UV irradiated sample (bottom). With the help of the surface potentials the local carrier concentration can be determined, Fig. 2(b) ^43^. The carrier concentrations near the surface for the UV-irradiated and non-irradiated samples are comparable, however, while the UV-irradiated sample exhibits a very shallow depth profile, the non-irradiated sample exhibits a rapid decrease within the first 4 *μ*m. In consequence, it can be inferred that the oxygen vacancy concentration profile resulting from low pressure annealing with UV-irradiation is markedly different from that for the samples similarly annealed but not exposed to UV-irradiation.

The observation from the non-UV-irradiated samples that the sheet carrier concentration decreased below the measurement limit after 50 days (Table 1) is consistent with the restriction of the initial carrier profile to the top few microns (Fig. 2(b)). Using the oxygen-vacancy diffusivity determined for STO by De Souza *et al*.^7^, it is found that after 50 days at room temperature the diffusion length of oxygen vacancies is ≈10 *μ*m. In consequence, the low pressure annealed sample not exposed to UV-irradiation can re-oxidize, returning to the insulating state, while the near constant depth dependent concentration of the UV-exposed sample is not detectably altered.

**Figure 3:**
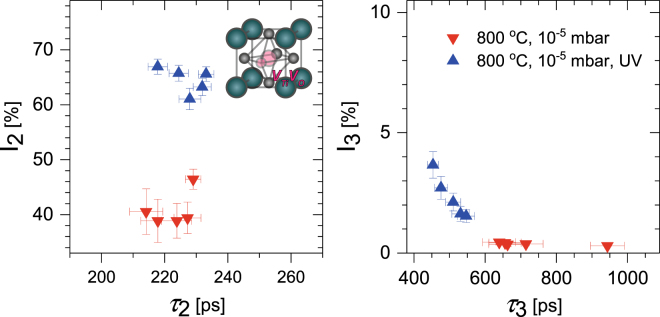
The two defect components obtained from three lifetime component free fits of the PALS spectra for positron implantation energies between 10 and 18 keV for a sample exposed to UV-irradiation during low pressure anneal (up triangle, blue), and a sample annealed but without UV exposure (down triangle, red). The inset shows a V_Ti_V_O_ vacancy complex.

To gain further insight on the defect content of the near surface region of the samples VEPALS measurements were performed using the *PLEPS instrument* on the neutron induced positron source (NEPOMUC) beamline at the *Heinz Maier*-*Leibnitz Zentrum Munich* research reactor^44,45^. The spectra were best fitted using three positron lifetime components, a reduced bulk lifetime and two vacancy-related defect components. The defect component results for positron implantation energies between 10 keV and 18 keV, which correspond to mean implantation depths varying between approximately 300 to 800 nm, are shown in Fig. 3. The second lifetime component was found to be approximately 225 ± 6 ps, this is in good agreement with the density functional theory (DFT) calculated value for the Ti-vacancy oxygen vacancy complex, V_Ti_V_O_, of 225 ps^46^. A significant increase in trapping to this defect was observed for the UV-irradiated low pressure annealed sample, compared to the sample annealed without UV-irradiation (samples stored in air for >50 days). The oxygen monovacancy is normally expected to be positively charged and hence not to trap positrons. If this was occurring a defect lifetime of approximately 160 ps would be expected^47^. The lifetime of the Ti vacancy in STO is approximately 180 ps, while that for the Sr vacancy is 280 ps^47^. The increase in the intensity of the V_Ti_V_O_ lifetime component observed here (Fig. 3) can only result from either an increase in the defect trapping coefficient, which would require a change of the charge state of the defect to a more negative value, or from an increase in the concentration of defects in the low pressure annealed sample exposed to UV-irradiation compared to the non-UV-irradiated sample. An increase in the concentration of V_Ti_V_O_ defects is consistent with an increase in the oxygen vacancy concentration in the UV-exposed sample.

Figure 3 also shows the results for the third lifetime component, this has negligible value for the non-UV-irradiated sample, but there is evidence of trapping to a vacancy cluster defect with an approximate lifetime of 500 ± 40 ps in the UV-exposed sample. Positron trapping vacancy cluster defects involved on the order of 10–20 vacancies have been previously observed in STO thin films^48^. Trapping to vacancy cluster defects in the UV-exposed sample supports the presence of an increase in oxygen vacancy defects compared to the non-UV-exposed sample.

## Discussion

Considering all the results described above we propose a mechanism by which the UV radiation of the plasma plume influences STO. The VEPALS measurements observe an increase in positron trapping to oxygen vacancy containing vacancy complexes providing evidence that an increase in oxygen vacancy concentration is responsible for the increase in conductivity. The very different depth dependent carrier concentration profiles obtained from the cross sectional SKPM provide further evidence for the presence of much higher concentration of oxygen vacancies within the volume of the UV irradiated sample compared to the non UV irradiated sample. The temperature and pressure environment for both samples was equal. Further the formation of interstitials in STO can be excluded restricting the formation of oxygen vacancies to the surface^6^. Hence we conclude that the UV irradiation enhances the oxygen vacancy incorporation rate at the surface. The oxygen vacancy incorporation can be described by Equation 1 ^5–8^ in the Kröger-Vink notation^49^.

1 $${{\rm{O}}}_{{\rm{O}}}^{x}+{{\rm{2h}}}^{\cdot }\rightleftharpoons \frac{1}{2}{{\rm{O}}}_{2}+{{\rm{V}}}_{{\rm{O}}}^{\cdot \cdot }$$

In the same way the incorporation of oxygen can be described by Equation 2 ^5–8^ in the Kröger-Vink notation^49^.

2 $$\frac{1}{2}{{\rm{O}}}_{2}+{{\rm{V}}}_{{\rm{O}}}^{\cdot \cdot }+2{{\rm{e}}}^{-}\rightleftharpoons {{\rm{O}}}_{{\rm{O}}}^{x}$$

The incorporation of oxygen (Equation 2) in reality is a multi step reaction including the adsorption of molecular oxygen, electron transfer, O-O bond dissociation and incorporation of atomic oxygen into oxygen vacancies. However, in the case of sufficient oxygen vacancies being present the electron transfer is considered as the rate limiting step^31,50^. Considering this, the known UV-enhanced oxygen incorporation rate for STO is under consideration of Equation 2 explained by additional electrons provided by the electron hole generation of the UV radiation (Equation 3)^29–32^.

3 $$nil+hv\to {{\rm{e}}}^{-}+{{\rm{h}}}^{\cdot }$$

In a similar fashion the hole transfer can be considered the rate limiting step for the incorporation of oxygen vacancies (Equation 1) in case of sufficient lattice oxygen being present. Considering Equation 3 the enhanced oxygen vacancy incorporation can be attributed to the generation of additional holes by the UV radiation. We can consequently explain the persistent photo conductivity in STO, which has been discussed in literature controversially^28^, by an increased oxygen vacancy incorporation rate triggered by the generation of additional holes. Considering Equations 1, 2 and 3 we can not only explain the oxygen vacancy incorporation by the UV irradiation of the plasma plume observed in our experiments, but we can equally explain the enhanced oxygen incorporation in presence of the plasma plume reported in literature^51,52^. This in turn explains, why no UV induced reduction was observed at 10^−3^ mbar. The absence of conductivity for samples processed at room temperature may be explained by the reoxidization of the UV induced oxygen vacancies upon air exposure, as they are restricted to the surface due to the limited kinetic at such temperatures^53^. It further shows that the holes and electrons generated by the UV radiation of the plume are not persistent and thus do at best introduce perishable conductivity. Only if they participate in the chemical reduction of the sample persistent photo conductivity seems observable.

## Conclusion

Summarizing, we have been able to separate the influence of the UV-irradiation accompanying the PLD laser plume on the oxide sample from the possible effects caused by the impinging growth species. STO samples were exposed to typically low pressure oxide film growth PLD conditions with and without an ignited plume. Our specially designed holder enabled samples to be prepared without being exposed to impinging species, but at the same time being exposed to the UV radiation. Marked differences in the carrier concentration profile and variation with time were observed. The UV-exposed samples demonstrated a high carrier concentration that was nearly constant with both depth and time. By contrast the non-UV-exposed STO samples showed a carrier concentration that decreased strongly with depth and time. The results suggest that the oxygen vacancy concentration is higher and that the profile with depth is markedly shallower in the UV-exposed samples compared to the non-UV-irradiated samples. The depth dependent positron lifetime measurements detect an increase in trapping to V_Ti_V_O_ divacancies in the UV-exposed sample consistent with an increased oxygen vacancy concentration. We have clearly shown that the UV radiation emitted by the plasma plume during pulsed laser deposition of oxide thin films plays a key role for their properties. The mechanism behind this was identified as a UV enhanced oxygen vacancy incorporation rate, thus also providing an explanation for persistent photo conductivity in STO reported in literature. Our studies identified the UV radiation of the plasma plume as a key influence factor on the redox-processes relevant for the oxidization state of oxide thin films and the underlying substrate during PLD.

## Methods

The base pressure in the used PLD system was 10^−8^ mbar and the excimer laser is a *Compex 205F* - *COHERENT*. An IR-Diode laser heater with a wavelength of 925 nm was used as heater. The Hall measurement system is a *Lakeshore 8400 Series*. The datasets generated during and/or analyzed during the current study are available from the corresponding author on reasonable request.
